# Engaging Posterior Capitellum Fracture and Elbow Posterolateral Rotatory Instability: Is It Always Necessary to Treat the Bone Defect?

**DOI:** 10.1155/2020/3260106

**Published:** 2020-02-26

**Authors:** Juan M. Patiño, Juan M. Torres Moirano

**Affiliations:** Departamento de Ortopedia y Traumatologia, Servicio de Cirugía de Miembro Superior, Hospital Militar Central “Cosme Argerich”, Buenos Aires., Argentina

## Abstract

**Case:**

We present a 23-year-old male with 7 episodes of left elbow dislocation during a two-year period. He had a positive pivot shift test with engaging. The original treatment plan included lateral ulnar collateral ligament reconstruction and eventually bone defect grafting and osteosynthesis. However, a bone graft was not performed. After 2 years of follow-up, the DASH score was 3.3. The Mayo Score was 90.

**Conclusion:**

Posterolateral instability associated with an engaging bone defect, in every elbow extension and pivot shift test, was not found in the literature. The capitellum defects are associated with PLRI and make it worse. Bone reconstruction may not be indicated.

## 1. Introduction

In 1966, Osborne and Cotterill described a posterior capitellum defect in recurrent elbow dislocation (Osborne-Cotterill lesion) [1–4]. Posterolateral rotatory instability (PLRI) was, more recently, described by O‘Driscoll in 1991 [5].

Impression fractures of the distal portion of the capitellum could contribute to the loss of radiocapitellar stability and may be unrecognized, especially in a dislocation fracture of the elbow [6].

Information about the treatment guides and outcomes of the Osborne-Cotterill fractures is scarce [2].

It is given by case reports and short series that show a lack of consensus.

Cases of posterolateral instability associated with an engaging bone defect, in every elbow extension and pivot shift test, were not found in the literature.

In this particular case that we are presenting, a question emerges during surgical planning. Could the instability be treated without fixing the engaging bone defect?

Therefore, we report a case in which the instability was treated without fixing the bone defect.

Informed consent was obtained from the patient included in the study, and the institutional Ethical Committee approved the retrospective medical chart review.

## 2. Case

We report a case of a 23-year-old male with a history of 7 episodes of left elbow dislocation during a two-year period. The first dislocation was the result of a bicycle fall with the arm in extension and mild supination. Treatment in another center included closed reduction and immobilization with the elbow in 90 degrees for 10 days. The other elbow dislocation episodes occurred while the patient performed daily activities; these dislocations reduced spontaneously. Daily activities were significantly limited by subjective instability and apprehension.

Our first examination showed elbow extension apprehension, a positive pivot shift test with engaging, audible clank, and pain in the lateral aspect of the elbow. Elbow medial ligaments remained stable during the stress (valgus and varus) tests. AP and lateral elbow x-rays did not show bone injuries. A pivot shift test was positive with engaging due to the bone defect, resulting in difficult reduction under fluoroscopy.

The preoperative Disabilities of the Arm, Shoulder, and Hand (DASH) score was 76.7 points, and the Mayo Elbow Performance Score was 5 points.

A left elbow CT scan showed a bone deficit at the posterior capitellum site. This finding appeared to be related with the erosion produced by the impact of the radial head in recurrent dislocations.

**Figure 1:**
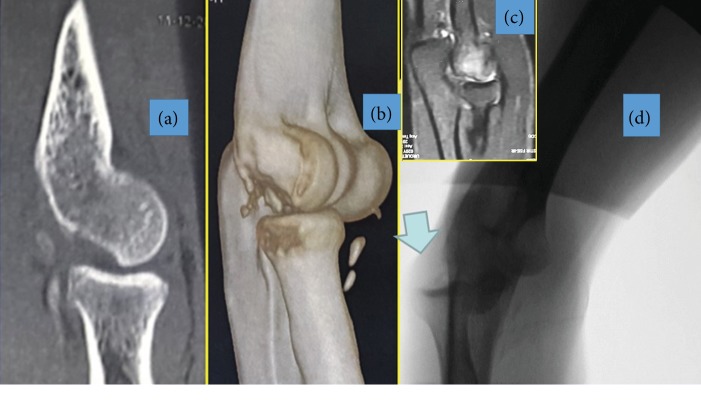
(a, b, and c) TC scan and MRI showing capitellum fracture. (d) Engaging pivot shift.

MRI revealed distal humerus bone edema (Figure 1).

The preoperative treatment plan included lateral ulnar collateral ligament reconstruction and eventually bone defect grafting and osteosynthesis. The examination under anesthesia and image intensifier confirmed posterolateral rotatory dislocation and engaging ([Supplementary-material supplementary-material-1]).

A Kocher incision of 8 centimeters long was performed. A lateral capsular and ligamentous deficit was observed. An autologous graft was taken from the palmaris longus tendon. Two holes were performed in the proximal ulna: one in the supinator crest tubercle and the other at 2 centimeters proximal to the former, close to the annular ligament insertion, keeping the bone bridge between them. The humerus hole was placed 2 millimeters anteriorly and distally from the lateral condyle. Using a tendon hook, the palmaris longus graft was passed through the holes.

Stability was tested. Intraoperative observation revealed that the posterior capitellum deficit was not implicated in the elbow excursion after lateral ligament reconstruction. Therefore, a bone graft was not performed. Elbow immobilization in 90 degrees and full pronation for 4 weeks were performed.

## 3. Results

After 3 weeks, active and passive flexion exercises were indicated. Then, progressive elbow extension was allowed, initially in 90 degrees of flexion and increasing 10 degrees of extension each week until the full extension was achieved in 8 weeks.

The DASH (Disabilities of the Arm, Shoulder, and Hand) score was used to evaluate the function of the elbow and symptoms during a 3-month period; the obtained score was 24.4 points. After 2 years of follow-up, the DASH score increased to 3.3. The Mayo Elbow Performance Score was 70 after 3 months and 90 after 2 years.

## 4. Discussion

We report a patient with unstable elbow and engaging bone defect. The joint was stabilized with no need of bone defect repair, and a favorable outcome was achieved. In this particular case that we are presenting, a question emerges during surgical planning. Could the instability be treated without fixing the engaging bone defect?

PLRI and ligament reconstruction have been well described [7].

In cases of bone defect associated with instability, some authors treat the instability but not the bone defect; some others treat both. Lateral elbow reconstruction has shown predictable results, but scarce information was found in the literature (cases reports and short series) about this kind of chronic impression fractures, even more when this bone defect is associated with elbow engaging.

The bone stock deficit in the posterior capitellum could be a significant indicator of PLRI. In 2008, a study based on images of the elbow reported a high prevalence (82%) of PLRI in injuries of LCL in patients with capitellum defect [1, 4].

Faber and King published in 1998 an isolated case of a 27-year-old female with capitellum fracture and PLRI but with no engagement. This defect was described as an impression of the posterior capitellum, and the treatment was only ligament reconstruction [8].

Capitellum height reduction decreases the tension of the LCL complex, potentially leading to instability.

In addition, changes in the structure of the capitellum could comprise joint stability due to the radiocapitellar congruence alteration. Ligament reconstruction techniques in addition to bone defect correction using bone graft were successful in patients with bone defect greater than one-fourth of the joint surface. The biomechanical evaluation could be important to determine the exact relevant amount of bone loss secondary to PLRI in order to establish the optimal surgical treatment for this patient population [2, 9].

Clark et al. described a case of capitellum fracture associated with PLRI. The patient was treated with osteochondral autograft and LCL ulnar reconstruction. The author stated that the elbow stability was inversely proportional to the size of the capitellum defect. But he emphasized that it is unknown what percentage of joint surface loss should be treated using surgical repair [10].

Shukla and O'Driscoll reported 2 cases: a 38-year-old female patient with a history of traumatic injury in extended arm resulting in impression fracture at the anterolateral radius head border associated with capitellum impression fracture (superior posterolateral aspect) and displaced avulsion of the supinator crest tubercle at the LCL ulna insertion site. Intraoperative examination revealed engaging elbow and subluxation with greater than 25-degree extension with the forearm in supination. It was not reported if the engaging appeared with each maneuver.

The article described the capitellum impression fracture reposition using cancellous bone graft. In the 2nd case, ligament reconstruction was performed, in a 35-year-old female with instability and depressed comminuted fracture involving the inferior aspect of the capitellum [11].

Many questions arise as regards the surgical intervention and evaluation of patients with posterolateral rotatory instability associated with posterior engaging fracture. Information is scarce; the options for distal humerus bone reconstruction appear to have pitfalls and uncertain results.

No studies were found describing what type of injuries leads to engaging the proximal radius in each trial of elbow extension and which of these injuries should be fixed. We consider that the bone defect size added to capsular insufficiency and laxity could result in this condition.

## 5. Conclusions

The capitellum defects are associated with PLRI and could make it worse. However, once the ligaments have been reconstructed, probably the mentioned defect could be excluded from the joint excursion. Consequently, bone reconstruction may not be indicated, since the procedure could lead to complications and the results are uncertain.

More studies are needed to determine in which bone defect (size localization and depth) the surgical treatment is necessary.

## Supplementary Materials

Supplementary Materials Video 1: positive, engaging pivot shift maneuver and fluoroscopy view under anesthesia.
